# Recent Advances in the Development of Noble Metal NPs for Cancer Therapy

**DOI:** 10.1155/2022/2444516

**Published:** 2022-01-28

**Authors:** Rui Zhao, Jia Xiang, Bo Wang, Lin Chen, Songwen Tan

**Affiliations:** Xiangya School of Pharmaceutical Sciences, Central South University, Changsha 410013, China

## Abstract

With the development of nanotechnology, noble metal nanoparticles are widely used in the treatment of cancer due to their unique optical properties, excellent biocompatibility, surface effects, and small size effects. In recent years, researchers have designed and synthesized a large number of nanomedicines that can be used for cancer treatment based on the morphology, physical and chemical properties, mechanism of action, and toxicological studies of noble metal nanoparticles. Furthermore, the integration of diagnosis and treatment, hyperthermia, cytotoxicity research, and drug delivery system based on the study of noble metal nanoparticles can be used as effective means for cancer treatment. This article focuses on the analysis of noble metal nanoparticles that are widely used in the treatment of cancer, such as gold nanoparticles, silver nanoparticles, platinum nanoparticles, and palladium nanoparticles. The various methods and mechanisms of action of noble metal nanoparticles in the treatment of cancer are objectively summarized in detail. Based on the research on the therapeutic safety and toxicity of noble metal nanoparticles, the development prospect of noble metal nanoparticles in the future clinical application is prospected.

## 1. Introduction

Cancer is the biggest enemy of human health at present. According to the global cancer statistics, the mortality rate caused by cancer in China is 24%, and with the increase in the number of patients, China spends more than 220 billion yuan per year on cancer [1, 2]. Drug therapy (chemotherapy) has a significant therapeutic effect on most tumors. As one of the main effective methods for the treatment of cancer, chemotherapy has significantly improved the survival and quality of life of cancer patients [3]. However, most of the antitumor drugs are nonspecific drugs with large toxic and side effects [4], which can cause drug resistance while seriously damaging the body function of patients [5]. Therefore, the improvement of antitumor drugs with poor water solubility, imperfect absorption in vivo, and low bioavailability has become a hotspot in the research and development of innovative drugs in the field of modern medical research [6].

**Figure 1:**
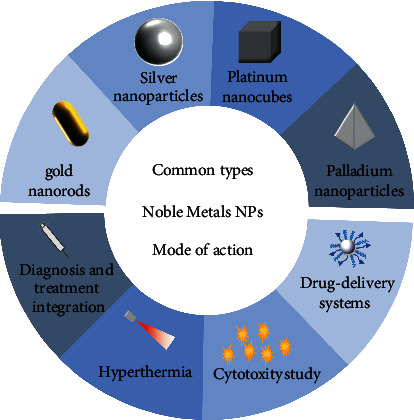
Types and mechanisms of noble metal nanoparticles for cancer therapy.

The emergence of nanodrugs effectively makes up for the shortcomings of traditional anticancer drugs. In 1964, nanoliposomes were first reported for the delivery of antitumor drugs. Researchers found that, compared with traditional antitumor drugs, nanoliposomes had more stable plasma concentration levels and lower toxic and side effects and were significantly superior to traditional drugs in pharmacokinetics and pharmacodynamics [7]. Since then, nano-antitumor drugs have developed rapidly and become a research hotspot due to their outstanding advantages such as controlled release, targeting, high efficiency, and low toxicity [8]. With the development of nanotechnology, noble metal nanoparticles have been widely used in the biomedical field due to their unique optical properties, excellent biocompatibility, surface effect, and small size effect. Based on the definition of nanoparticles, noble metal nanoparticles are defined as particles smaller than 100 nm in size in at least one dimension [9]. This paper focuses on the noble metal nanoparticles commonly used in the treatment of cancer, including gold nanoparticles, silver nanoparticles, platinum nanoparticles, and palladium nanoparticles, and summarizes the various methods and mechanisms of noble metal nanoparticles in the treatment of cancer in detail, which are briefly described in Figure 1.

## 2. Types of Noble Metal Nanoparticles for the Treatment of Cancer

The three-dimensional size of noble metal nanoparticles has at least one-dimensional distribution within 100 nm [10]. Noble metal nanoparticles are widely used in biomedicine because of their large specific surface area, excellent biological properties, easy preparation, high efficiency, and low toxicity. At present, the noble metal nanoparticles commonly used in the treatment of tumors mainly contain gold, silver, platinum, copper, lead, and other noble metal materials. Through different synthesis conditions, spherical, rod-like, powder, film, and other noble metal nanoparticles with different morphologies can be prepared, such as the chemical method based on the liquid phase sol-gel method [11], as well as laser ablation [12], vacuum sputtering [13], vacuum evaporation method [14], ion implantation method [15], and other physical methods. When the morphology, size, and composition of noble metal nanoparticles change, their physical and chemical properties also change accordingly [16]. For example, spherical gold nanomaterials usually have strong absorption spectra in the visible region, while rod-shaped gold nanomaterials have strong absorption spectra in the near-infrared region. This phenomenon is mainly related to the localized surface plasmon resonance (LSPR) effect of noble metal nanoparticles [17]. Meanwhile, in the LSPR region, the surface-enhanced Raman scattering (SERS) effect [18] and metal-enhanced fluorescence (MEF) effect [19] are also found on the surface of noble metal nanoparticles, which contribute to the unique electronic properties and efficient photothermal conversion of noble metal nanoparticles.

### 2.1. Gold Nanoparticles

**Figure 2:**
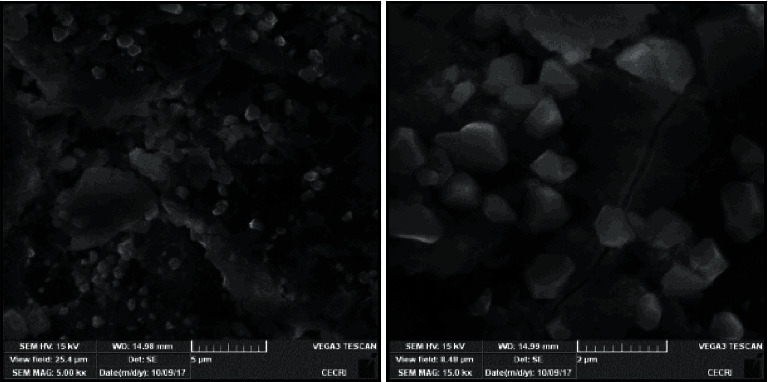
SEM images of gold NPs capped with *Croton caudatus* Geisel leaf extract (Vijaya Kumar et al. [28]).

In recent years, based on the excellent properties of gold nanoparticles, the synthesis and application of gold nanoparticles (GNPs) have become a research hotspot. At present, the efficient methods commonly used for the preparation of gold nanoparticles are the electrochemical method [20], two-phase method [21], and seed growth method [22, 23]. In addition, the biosynthesis method [24] is also a new method commonly used for the preparation of gold nanoparticles with good monodispersity and small particle size. With gold as the matrix, different gold nanostructures can be synthesized based on different synthesis methods and different synthesis processes. Common structures of gold nanomaterials include gold nanorods [25], gold nanoprisms [26], gold nanocages [27], etc. The SEM images of gold NPs capped with *Croton caudatus* Geisel leaf extract obtained by Vijaya Kumar et al. [28] are shown in Figure 2.

Gold nanoparticles are widely used in biomedical fields due to their small particle size, good monodispersity, high tissue permeability, and colloidal stability [29]. They can be used to treat tumors by inhibiting angiogenesis, hyperthermia, and loading antitumor drugs. Studies have successively found that GNPs can act on heparin-binding proteins, such as basic fibroblast growth factor (bF-GF) and vascular endothelial growth factor-165 (VEGF-165) [30], epidermal growth factor receptor (EGFR), and vascular endothelial growth factor receptor-2 (VEGFR-2) [31]; this limits the growth and metastasis of the tumor cells. In contrast, GNPs have no inhibitory effect on non-heparin-binding protein growth factors. On the other hand, GNPs have an LSPR effect, which can effectively absorb light energy in the near-infrared region and convert it into heat energy. Hyperthermia is performed in the tumor site to induce protein denaturation and cell apoptosis, thereby killing the cancer cells [32]. In recent years, the design and synthesis of antitumor drugs that can be used for photothermal therapy have become a research hotspot with gold nanorods (or gold nanospheres) as the matrix and functionalized modification on its surface [33]. Song et al. [34] reported the gold nanorod vesicles (rGO-AuNRVes) loaded with mixed reduced graphene oxide (rGO) and doxorubicin (DOX). The experimental results show that rGO-AuNRVes have excellent photoacoustic (PA) properties and photothermal effects, realizing the combination of hyperthermia and targeted drug delivery. At the same time, gold nanoparticles can also be used as drug carriers, directly or after surface modification combined with drugs through covalent bonds [35] or noncovalent bonds [36], to load a variety of drugs to reach the target site, improve the bioavailability of drugs, and realize the controlled release of drugs. Sun et al. [37] used gold nanorods as the carrier, surface modified with polyethylene glycol (PEG) and 64Cu, and used positron emission tomography (PET) for optical imaging. This drug delivery system showed high targeting ability, which could realize individualized medication and avoid side effects.  Table 1 lists the common drug carrier systems using gold nanoparticles for clinical cancer therapy in recent years. In addition, radiation sensitization [45], excellent biocompatibility, and unique optical properties [46] have promoted the integrated development of diagnosis and treatment of gold nanoparticles. However, as a kind of noble metal medical material for cancer therapy, researchers should pay attention to its biological safety and environmental safety. At the same time, the limited reserves and expensive prices limit the research and utilization of gold nanoparticles. The pursuit of economic security, high efficiency, and low toxicity of gold nanomaterials is still the key point of future research [37, 47–53].

### 2.2. Silver Nanoparticles

**Figure 3:**
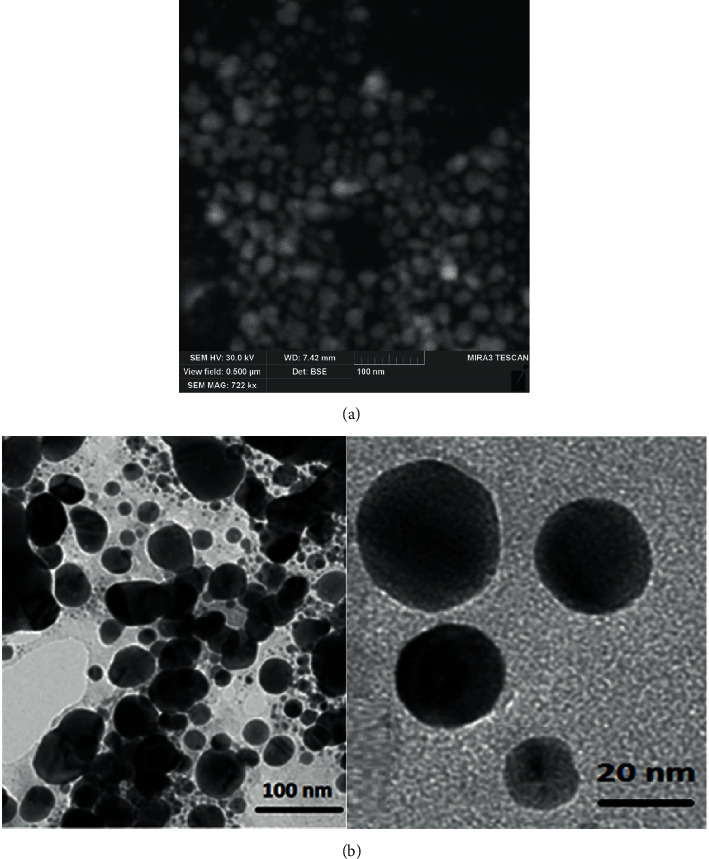
(a) SEM of silver nanoparticles obtained under plasma treatment and sodium alginate conditions (C_AgNO3_3.0 mmol/l) (Skiba et al. [56]). (b) TEM of silver nanoparticles obtained under plasma treatment and sodium alginate conditions (C_AgNO3_3.0 mmol/l) (Skiba et al. [56]).

Compared with gold nanoparticles, the stability of silver nanomaterials (AgNPs) is slightly poor and they will be oxidized gradually in oxygen-containing fluids [54]. However, based on the excellent characteristics of the LSPR effect and SERS effect, strong antibacterial activity, and catalytic performance of AgNPs, AgNPs have become the most widely studied inorganic nanomaterials. One of the most innovative and environmentally friendly green methods to produce silver nanoparticles is low-temperature nonequilibrium contact [55, 56]. Skiba et al. [56] obtained SEM images (Figure 3(a)) and TEM images (Figure 3(b)) of silver nanoparticles by using low-temperature nonequilibrium contact plasma and stabilizer polysaccharide (sodium alginate). At present, common silver nanostructures include silver nanowires, silver nanocubes, and silver nanospheres [57]. Ideal optical properties can be obtained by adjusting the size, morphology, and structure of silver nanoparticles [58]. Bian et al. [58] cleverly controlled the size and morphology of silver nanoparticles by changing the volume of silver nitrate (AgNO_3_) added. This kind of peptide-directed hierarchical mineralized silver nanocages prepared by biomaterial template-directed mineralization has a high tumor-killing rate of 82.7% and a photothermal conversion rate of 46.1%, which belongs to the most powerful photothermal conversion of silver nanomaterials.

AgNPs have cytotoxicity, second only to mercury in antibacterial effect, and have the advantages of safety, slow release, and broad-spectrum antibacterial. The use of silver as a bactericidal material for the treatment of tumors has become a research hotspot since the 1990s. It is worth noting that the toxicity of AgNPs is not a direct effect of free Ag^+^ but is caused by the oxidative stress of Ag nanoparticles [59]. Gurunathan et al. [60] found that AgNPs can induce oxidative stress by producing reactive oxygen species (ROS) in cells, leading to cytotoxicity, apoptosis, and necrosis of cancer cells, which is a new step in the treatment of tumors by using the antibacterial activity of AgNPs. Shi et al. [61] used doxorubicin (DOX) as a drug model, AgNPs were deposited on graphene oxide (GO) by hydrothermal reaction, and then GO@Ag was functionalized by DSPE-PEG2000-NGR to prepare Go@Ag-DOX-NGR. The results show that Go@Ag-DOX-NGR has the ability of lesion targeting, photothermal ablation, and excellent X-ray imaging in the diagnosis of tumors, indicating that AgNPs have great potential in tumor diagnosis and treatment.

### 2.3. Platinum Nanoparticles

**Figure 4:**
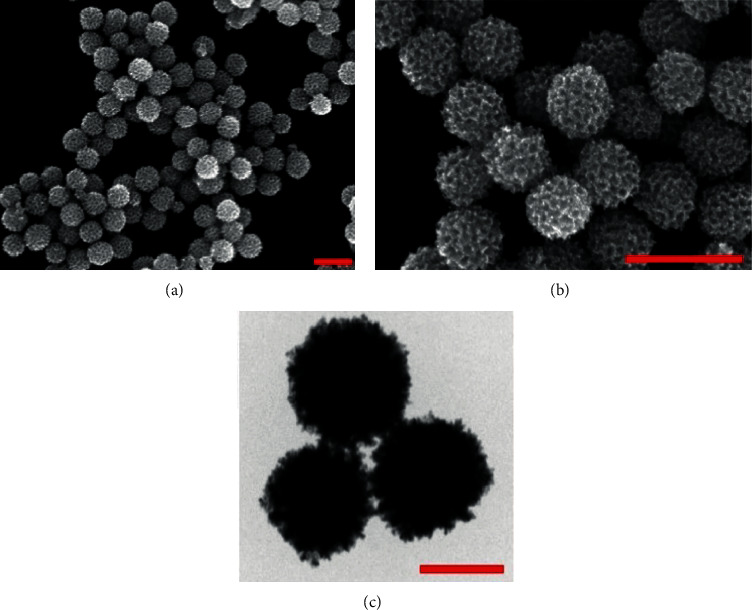
(a). Low-magnification SEM imaging of mesoporous platinum nanoparticles (Fu et al. [71]). (b). High-magnification SEM imaging of mesoporous platinum nanoparticles (Fu et al. [71]). (c). TEM imaging of mesoporous platinum nanoparticles (Fu et al. [71]).

Since scientists first discovered the antitumor effect of cisplatin in 1969 [62], the use of metal complexes in the treatment of tumors has attracted the wide attention of medical workers. Based on the research of cisplatin, gold, silver, platinum, rhodium, palladium, and other noble metal complexes have been synthesized, which can be used in the diagnosis and therapy of cancer. Organoplatinum compounds belong to the biological alkylating agents in antitumor drugs [63], which can induce apoptosis and necrosis of tumor cells by changing the structure of target DNA and inhibiting the cell cycle [64]. Studies have shown that platinum metal nanoparticles can enhance the antitumor efficacy by regulating a variety of signal channels and activating the immune system [65–67]. Platinum nanoparticles have been widely used in clinical applications due to their excellent catalytic performance [68], imaging ability [69], excellent photothermal conversion ability, and radiosensitization ability [70]. Fu et al. [71] performed low-magnification (Figure 4(a)) and high-magnification SEM imaging (Figure 4(b)) and TEM imaging (Figure 4(c)) on mesoporous platinum nanoparticles. The structure of platinum nanoparticles and indications in the treatment of tumors are shown in Table 2.

Cisplatin, as the first generation of platinum-based broad-spectrum antitumor drugs, has been one of the most widely used antitumor drugs since 1978. It is widely used in the clinical application treatment of prostate cancer, breast cancer, and bladder cancer [78, 79]. Cisplatin inhibits DNA replication of tumor cells, thereby hindering cancer cell division and inducing apoptosis [80]. Platinum nanoparticles combined with gemcitabine and other antitumor-targeted drugs can be used as a potential drug for the treatment of ovarian clear cell carcinoma (OCCC) [81] or the treatment of advanced pancreatic cancer [82]. It not only has synergistic and immunosuppressive effects but also has no cross-resistance. However, cisplatin can only be administered by injection and has short half-life, and long-term use will produce drug resistance and serious toxicity [83].

The second-generation broad-spectrum antitumor drugs carboplatin and nedaplatin developed based on cisplatin have significantly improved their antitumor efficacy. Carboplatin is mainly used for the treatment of ovarian cancer and melanoma in the clinic. Its physicochemical properties, mechanism of action, antitumor spectrum, and anticancer activity are similar to those of cisplatin, and its nephrotoxicity, ototoxicity, and gastrointestinal side effects are weakened. But the administration is still intravenous administration and accompanied by serious bone marrow suppression and anemia and other adverse reactions [73]. The pharmacokinetic characteristics of nedaplatin are similar to those of carboplatin. Compared with cisplatin, its toxicological characteristics are significantly improved. Nedaplatin is mainly used for the treatment of lung cancer, esophageal cancer, and head and neck cancer in the clinic [84, 85].

The third-generation antitumor drugs include oxaliplatin, lobaplatin, and heptaplatin. Compared with cisplatin and carboplatin, oxaliplatin has specific targets, mechanisms of action, and drug resistance mechanisms [86]. Oxaliplatin can be used in combination with other antitumor drugs for the treatment of advanced colorectal cancer with superior efficacy and without cross-resistance. It also has an obvious inhibitory effect on drug-resistant strains of cisplatin and carboplatin [87]. The antitumor mechanism of lobaplatin is similar to that of cisplatin. Its antitumor efficiency is significantly improved, and its toxicity is similar to that of carboplatin. The main toxicity of lobaplatin is bone marrow hematopoietic inhibition, and its renal toxicity is low [76]. Heptaplatin combined with 5-fluorouracil (5-FU) can be used for the treatment of advanced cancer, but it will produce slight and reversible proteinuria toxicity [77].

The versatility and high selectivity of platinum nanoparticles make them a promising candidate in the field of cancer therapy [88]. However, platinum compounds often cause acute kidney injury (AKI) during treatment [89]. In addition, many adverse reactions such as dose limitation, systemic toxicity, and cross-resistance limit their clinical application. Therefore, it is urgent to prepare high-efficiency, low-toxicity, safe, and stable platinum metal nanoparticles and their complexes.

### 2.4. Palladium Nanoparticles

**Figure 5:**
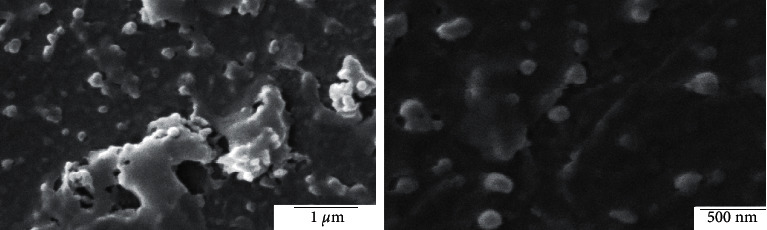
SEM micrographs of palladium NPs exposed to *C*. *vulgaris* extract (Arsiya et al. [91]).

Palladium nanomaterials are often used as substitutes for gold nanoparticles in the treatment of tumors because of their excellent photothermal stability, excellent catalytic activity, and significant price advantages [90]. Arsiya et al. [91] performed SEM scanning of palladium nanoparticles exposed to *C. vulgaris* extract, and the image is shown in Figure 5. The two-dimensional structure of ultrathin hexagonal palladium nanosheets prepared by Huang et al. [92] showed high stability under near-infrared region light. And compared with gold/silver nanostructures, palladium nanoparticles could effectively improve the photothermal conversion ability and biocompatibility. Studies have shown that the use of polymers to functionalize the surface of palladium nanoparticles can also significantly improve their biocompatibility, water dispersion, and physiological stability [90]. Bharathiraja et al. [90] modified palladium nanoparticles with chitosan (COS) and then functionalized with RGD peptide to prepare Pd@COS-RGD. The experimental results showed that the functionalized palladium nanoparticles can be used as an ideal nano-inorganic material for near-infrared region laser imaging and tumor diagnosis. The surface functionalization of palladium nanoparticles can significantly improve the catalytic activity and utilization rate, and the preparation method determines the performance of palladium nanoparticles. At present, the preparation methods of palladium nanoparticles mainly include the chemical reduction method [93], biological reduction method [94], and so on. As an efficient and mature traditional method, the chemical reduction method is often combined with other preparation methods. For example, Roy et al. [95] developed a one-pot synthesis method, in which polyvinyl alcohol (PVA) was used as the steric hindrance stabilizer and citric acid was used to reduce palladium chloride to design and synthesize high dispersion and low catalytic activity, which can be widely used in biomedical fields. The mature preparation methods, significant price advantages, and excellent optical properties determine that palladium nanomaterials have great potential for research and application in the field of cancer treatment.

## 3. Methods of Cancer Therapy Based on Noble Metal Nanoparticles

With the vigorous development of nanotechnology, the research on the physicochemical properties, toxicology, and mechanism of action of noble metal nanoparticles in the treatment of tumors has become more and more thorough. Based on the LSPR effect [17], the SERS effect [18], and the MEF effect [19] of noble metal nanoparticles' surfaces, noble metal nanoparticles can be used for the early diagnosis of tumor lesion locations. Based on the efficient photothermal conversion ability of noble metal nanoparticles, tumor tissues can be thermally killed. Based on the cytotoxicity of noble metal nanoparticles, the antibacterial and antitumor effects are obvious. Based on the characteristics of noble metal nanoparticle targeting and easy modification, a targeted drug delivery system can be constructed. Noble metal nanoparticles have shown great potential development prospects in the biomedical field of tumor treatment.

### 3.1. Diagnosis and Treatment Integration

The study has shown that the low diagnostic rate of early cancer may be the main reason for the high mortality of cancer patients in China [2]. Because the early stage of cancer is more hidden, diagnosis is difficult, and cancer cells are easy to spread through lymphatic vessels and other channels, leading to high cancer mortality. If tumor cells are found in the early stage of cancer, it will effectively delay the survival of patients. Therefore, it is of great significance to develop a material that can assist in diagnostic imaging. Noble metal nanoparticles have an LSPR effect, which leads to obvious optical absorption in a specific wavelength region. In addition, noble metal NPs have a nonlinear optical response with electric field intensity, which presents high sensitivity and obvious imaging contrast [96]. In addition, noble metal nanoparticles are small in size, are easy to be modified by a variety of groups, and have excellent biocompatibility. Therefore, noble metal nanoparticles can be used as contrast agents for in vivo imaging for detection, labeling, in vivo imaging, and disease diagnosis of early cancer lesions [97]. Studies have shown that the binding capacity of GNPs to cancer cells is 600 times that of normal cells [98]. Compared with normal cells, tumor cells are covered with more epidermal growth factor receptors (EGFRs). Specific antibodies are modified to the surface of noble metal nanoparticles to target tumor cells for early diagnosis and imaging and to realize tumor visualization. Zhao et al. [99] prepared a novel multifunctional magnetic silver nanocomposite Fe_3_O_4_/Ag, which can be conjugated to an epidermal growth factor receptor-specific antibody (C225) as a high-sensitivity tracer and a potential radiosensitizer. Later, the magnetic resonance phenomenon is used for diagnosis. Using the noble metal nanoparticles can realize the real-time monitoring and evaluation of drug delivery processes and treatment status [100]. At the same time, high-sensitivity and high-resolution imaging technologies such as optical imaging (OI), X-ray computed tomography (CT), and magnetic resonance imaging (MRI) can be used to realize the integration of modern medical diagnosis and treatment [101].

### 3.2. Hyperthermia

**Figure 6:**
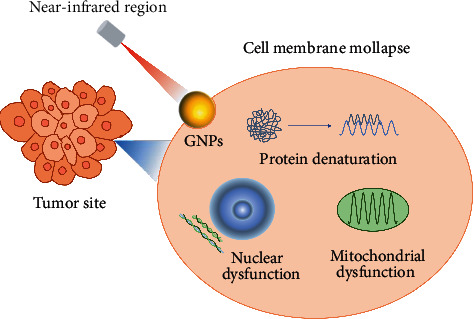
Apoptosis of tumor cells induced by hyperthermia.

Due to the rapid growth of the tumor site, the lack of lymphatic vascular reflux, vascular distortion, and aggregation, and slow blood flow velocity, the heat resistance of the tumor site is poor. Studies have shown that an elevated ambient temperature in tumor tissue can induce apoptosis of tumor cells [85]. Noble metal nanoparticles are based on this principle to achieve the purpose of cancer therapy. Compared with other bands, the light in the near-infrared region with a wavelength between 650 nm and 900 nm has a deep penetration depth of thermal damage to the tumor site. Therefore, the near-infrared region is usually preferred for in vivo tumor hyperthermia [102]. Under near-infrared laser irradiation, noble metal nanoparticles are enriched in targeted tumor sites and convert light energy into heat energy. High temperature promotes protein inactivation and causes irreversible damage to the cell membrane, thus killing cancer cells [103]. At the same time, hyperthermia promotes the further release of anticancer drugs and realizes the combination of chemotherapy and hyperthermia. The process of hyperthermia-induced tumor cell apoptosis is shown in Figure 6. Wang et al. [32] used gold nanoparticles to modify the reductive and pH double-sensitive polymer vesicles wrapped with DOX and realized the combination therapy of photothermal therapy and chemotherapy under near-infrared laser irradiation, which greatly improved the synergistic effect and drug utilization. Nowadays, hyperthermia can be divided into photothermal therapy (PTT) [104] and photodynamic therapy (PDT) [105]. Compared with traditional chemotherapy or radiotherapy, the most prominent advantages of hyperthermia are local targeted heating and specific selection of healthy tissues, which minimizes the damage to surrounding healthy tissues [106]. Smirnov et al. [107] reported the synthesis of core-shell noble metal anisotropic nanoparticles modified with cyanine-class fluorophores and stilbene-based Raman reporters. In perspective, it may be used for optical diagnostics and therapeutic hyperthermia. In addition, hyperthermia can also activate the immune system and enhance the immune ability of the system. The advantages of safety and specificity also make hyperthermia a promising treatment for tumors in clinical applications [108].

### 3.3. Cytotoxicity Study

**Figure 7:**
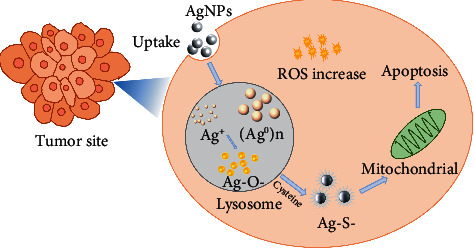
Mechanism of noble metal nanoparticles promoting apoptosis of tumor cells by cytotoxicity.

Silver nanoparticles have an excellent antibacterial effect, with cytotoxicity and heavy metal toxicity second only to mercury [109]. Since the 1990s, the use of silver as a bactericidal material for the treatment of tumors has become a research hotspot. Studies have shown that silver ions are more cytotoxic than other ions [110]. Toxicological studies in vivo and in vitro have shown that silver nanoparticles induce oxidative stress in cells to produce cytotoxicity by producing ROS. At the same time, oxidative stress can lead to mitochondrial membrane instability [111], cell apoptosis, or necrosis [112], thereby inhibiting tumor cells. The mechanism of noble metal nanoparticles promoting tumor cell apoptosis by cytotoxicity is shown in Figure 7. Foldbjerg et al. [113] explored the effects of silver ribosomes coated with Ag^+^ and polyvinylpyrrolidone on the human alveolar cell line A549. In the study, it was found that the treatment of antioxidant N-acetyl-cysteine significantly reduced the cytotoxicity of the two silver compounds, which further proved that AgNPs were the medium of ROS genetic toxicity. Studies have shown that the cytotoxicity of silver nanoparticles is closely related to their size and shape [59]. Compared with 50–100 nm-diameter nanoparticles, silver nanoparticles with the smallest diameter of 10–20 nm have the strongest cytotoxicity. Silver nanowires (1.5–25 *μ*m in length, 100–160 nm in diameter) are more toxic than spherical nanowires (30 nm in diameter) [59].

In addition, cisplatin is also a representative drug for cytotoxicity research. Cisplatin selectively binds to tumor cell DNA, blocks cell cycle, and inhibits DNA replication and transcription of tumor cells, thereby exerting cytotoxicity [114]. Shiny et al. [115] found that platinum nanoparticles can upregulate the expression of apoptotic markers and downregulate the expression of antiapoptotic genes to promote apoptosis and necrosis of tumor cells. Studies have shown that platinum nanoparticles have a good therapeutic effect on tumors.

### 3.4. Drug Delivery System

**Figure 8:**
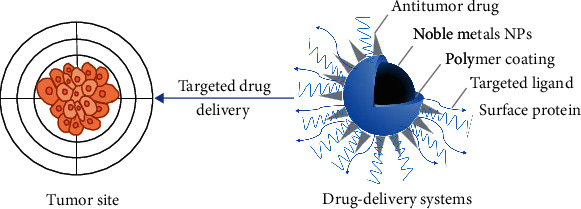
Brief schematic of drug delivery systems.

Antitumor drug delivery systems are constructed by physical embedding, covalent binding, and electrostatic interaction of antitumor drugs and noble metal nanoparticles [116]. Significantly, noble metal nanocarriers are based on the concept of targeting drug delivery systems (TDDSs) [117] and composed of noble metal-organic frameworks (NMOFs) with nanoparticle sizes of nm. As shown in Figure 8, gold nanoparticles, which are widely used in clinical practice, can be loaded with antitumor drugs [35], targeting ligands [42], and other drugs [43] by functional modification [33] or chemical bond binding [35, 36]. Ding et al. [35] designed and synthesized thiol-terminated PEGylated paclitaxel derivatives by the chemical synthesis method and conjugated with gold nanoparticles, which significantly improved drug loading, drug cycle time, and tumor cell killing efficiency. Noble metal nanoparticles not only have excellent biocompatibility, large specific surface area, liquid fluidity, and strong magnetic properties of solid [41] but also are sensitive to small changes in the external environment (pH, temperature, light, etc.) [118]. Therefore, a photo-responsive drug delivery system for noble metal nanoparticles can be established through remote spatiotemporal control. You et al. [119] designed and synthesized multifunctional doxorubicin (DOX)-loaded hollow gold nanoparticles (DOX@HAuNPs). Under the condition of near-infrared laser irradiation, GNPs were used as carriers to achieve targeted transport, accelerate the release of drugs, realize the combination of hyperthermia and chemotherapy, increase the anticancer activity of drugs, and reduce organ toxicity. Based on enhanced permeability and retention (EPR) [120], after the drug delivery system is phagocytized by macrophages, it can achieve passive targeted delivery [121] or achieve targeted delivery based on active targeted delivery [122] and environmental-responsive targeted delivery [118, 123–125] to achieve high efficiency and low toxicity, controlled release, and targeted delivery. However, the current research on antitumor drug nanocarriers mostly focuses on the basic theory, and the synthesis of complex nanocarrier drug technology is not yet mature. The low proportion of drugs that can be used in clinical experiments and practical applications, poor penetration ability, and low drug utilization limit the wide application of nano-antitumor drug carriers [126, 127].

## 4. Conclusion and Prospect

At present, cancer treatment still faces many challenges. But with the development of nanotechnology, the application of noble metal nanoparticles is expected to become an excellent weapon for the treatment of tumors. Noble metal nanoparticles have shown great potential in the biomedical field. The application of noble metal nanoparticles effectively overcomes the shortcomings of traditional tumor treatment methods and truly creates a new, safe, and minimally invasive tumor treatment system. In the diagnosis of cancer, based on the unique optical properties and excellent biocompatibility, researchers use advanced imaging technology to make noble metal nanoparticles as a contrast agent for self-imaging in vivo, which can significantly improve the signal intensity and enhance the contrast between normal tissues and tumor sites, to achieve the integration of diagnosis and treatment [97]. In the treatment of cancer, noble metal nanoparticles have SERS optical activity, which can efficiently convert light energy into heat energy, to carry out PTT or PDT on tumor sites [46]. In addition, noble metal nanoparticles can produce ROS to induce oxidative stress in cells to produce cytotoxicity and promote apoptosis and necrosis of tumor cells, thereby inhibiting the growth and reproduction of tumor cells [128]. At the same time, noble metal nanoparticles can also load a variety of antitumor drugs, respond to small changes in the external environment, and establish targeted drug delivery systems by passive and active transport, greatly improving the bioavailability of drugs [129]. However, noble metal nanoparticles also face many challenges in the application of clinical treatment. Noble metal nanoparticles are closely related to the health and life of patients. Therefore, great attention should be paid to their safety and toxicity. According to its biodynamics (absorption, distribution, metabolism, excretion) to evaluate and discover, noble metal nanoparticle drugs have fewer biocompatibility and biosafety issues [130]. The toxicity of nanoparticles may be different depending on the specific nanoproperties from which their reactivity, retention time, and distribution in the human body derive [131]. It is relatively difficult to determine the toxicity of precious metal nanoparticles based on the current toxicological studies. However, experimental animal trials have advantages, with one of the important ones being the assessment of the kinetics of nanoparticles through absorption, distribution, metabolism, and excretion (ADME) [132]. Li et al. [49] believed that the toxicity of noble metal nanoparticles should be different from the toxicity of antitumor drugs loaded on the surface of noble metal nanoparticles. Moreover, the oxidation state of noble metal nanoparticles should be also paid attention. In addition, due to the differences in the progress of theory and practice, and the difficulty in synthesizing complex nanomedicines, it is still necessary to further study the preparation, in vivo behavior, safety, and drug utilization of noble metal nanoparticles. The in-depth study on the mechanism of action, metabolism in vivo, pharmacokinetics, and toxicological principles of noble metal nanoparticles can promote the joint and synergistic effect of various mechanisms of noble metal nanoparticles, create new design ideas for the realization of high efficiency and low toxicity of noble metal nanocomposites, and lay a solid foundation for the exploration and development of new resources of noble metal nanoparticles.

## Figures and Tables

**Table 1:** Clinical application of gold nanoparticles as drug carriers.

Types of drug loading	Medicines vocabularies	Structural formula/molecular formula	Application/effect	References
Antitumor drugs	Oxaliplatin	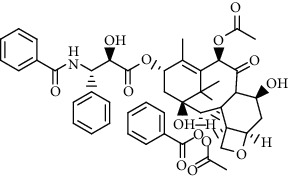	Loading drugs to improve water solubility	[35]
	Cisplatin compounds	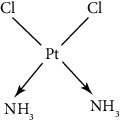	Establishment of drug delivery system with high efficiency and low toxicity	[38]
	Oxaliplatin	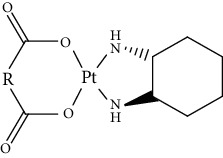	Establishment of drug delivery system with delayed control and targeting	[39]
	Chloroquine	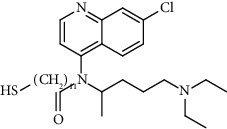	Establishment of stimuli-responsive drug release system to enhance the anticancer activity of drugs	[40]
Targeting ligand	Transferrin (TRF)	C_75_H_121_N_23_O_28_S	Overcoming traditional drug toxicity, immunogenicity, and poor integration	[41]
	Folic acid	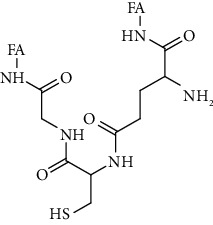	Enhancement of folate receptor expression level	[42]
Other substances	Protoporphyrin	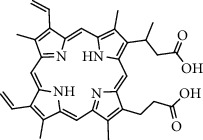	Photosensitizer for photodynamic therapy	[43]
	Tiopronin	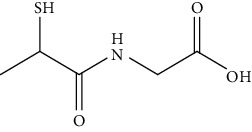	Significant repair of acute liver injury	[44]

**Table 2:** Structure of platinum nanoparticles and indications in tumor therapy.

Types of drugs	Structural formula	Mechanism of action	Adaptive diseases	References
Tolfplatin (cisplatin)	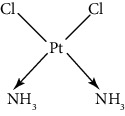	A COX-2 inhibitor that suppresses the production of PEG2	Breast cancer	[72]
Carboplatin	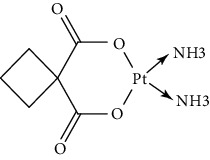	A COX-2 inhibitor with amino-functionalized polyphosphazene vesicles	Carcinoma of ovary	[73]
Nedaplatin	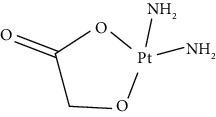	A COX-2 inhibitor with higher plasma concentrations of 5-fluorouracil	Esophageal squamous cell carcinoma	[74]
Lipoxal (oxaliplatin)	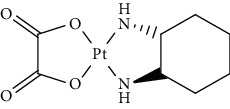	An inhibitor of DNA synthesis that prevents DNA replication and transcription and causes cell death	Gastrointestinal cancers	[75]
Lobaplatin	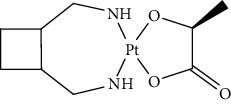	Inhibit the proliferation of cancer cells and promote apoptosis	Small-cell carcinoma	[76]
Heptaplatin	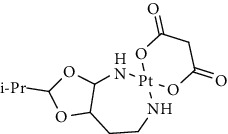	An inhibitor of DNA synthesis that prevents DNA replication and transcription	Gastric cancer	[77]

## Data Availability

All data supporting this work are included within the paper.
